# Multiple Inflammatory Biomarker Detection in a Prospective Cohort Study: A Cross-Validation between Well-Established Single-Biomarker Techniques and an Electrochemiluminescense-Based Multi-Array Platform

**DOI:** 10.1371/journal.pone.0058576

**Published:** 2013-03-05

**Authors:** Bas C. T. van Bussel, Isabel Ferreira, Marjo P. H. van de Waarenburg, Marleen M. J. van Greevenbroek, Carla J. H. van der Kallen, Ronald M. A. Henry, Edith J. M. Feskens, Coen D. A. Stehouwer, Casper G. Schalkwijk

**Affiliations:** 1 Department of Internal Medicine, Maastricht University Medical Centre, Maastricht, The Netherlands; 2 Nutrition and Toxicology Research Institute Maastricht (NUTRIM), Maastricht University, Maastricht, The Netherlands; 3 Top Institute Food and Nutrition (TIFN), Wageningen, The Netherlands; 4 Department of Clinical Epidemiology and Medical Technology Assessment (KEMTA), Maastricht University Medical Centre, Maastricht, The Netherlands; 5 Care and Public Health Research Institute (CAPHRI), Maastricht University, Maastricht, The Netherlands; 6 Cardiovascular Research Institute Maastricht (CARIM), Maastricht University, Maastricht, The Netherlands; 7 Division of Human Nutrition, Wageningen University, Wageningen, The Netherlands; Morehouse School of Medicine, United States of America

## Abstract

**Background:**

In terms of time, effort and quality, multiplex technology is an attractive alternative for well-established single-biomarker measurements in clinical studies. However, limited data comparing these methods are available.

**Methods:**

We measured, in a large ongoing cohort study (n = 574), by means of both a 4-plex multi-array biomarker assay developed by MesoScaleDiscovery (MSD) and single-biomarker techniques (ELISA or immunoturbidimetric assay), the following biomarkers of low-grade inflammation: C-reactive protein (CRP), serum amyloid A (SAA), soluble intercellular adhesion molecule 1 (sICAM-1) and soluble vascular cell adhesion molecule 1 (sVCAM-1). These measures were realigned by weighted Deming regression and compared across a wide spectrum of subjects’ cardiovascular risk factors by ANOVA.

**Results:**

Despite that both methods ranked individuals’ levels of biomarkers very similarly (Pearson’s r all≥0.755) absolute concentrations of all biomarkers differed significantly between methods. Equations retrieved by the Deming regression enabled proper realignment of the data to overcome these differences, such that intra-class correlation coefficients were then 0.996 (CRP), 0.711 (SAA), 0.895 (sICAM-1) and 0.858 (sVCAM-1). Additionally, individual biomarkers differed across categories of glucose metabolism, weight, metabolic syndrome and smoking status to a similar extent by either method.

**Conclusions:**

Multiple low-grade inflammatory biomarker data obtained by the 4-plex multi-array platform of MSD or by well-established single-biomarker methods are comparable after proper realignment of differences in absolute concentrations, and are equally associated with cardiovascular risk factors, regardless of such differences. Given its greater efficiency, the MSD platform is a potential tool for the quantification of multiple biomarkers of low-grade inflammation in large ongoing and future clinical studies.

## Introduction

Biomarker measurements representing low-grade inflammation have gained increasing importance in the management and understanding of cardiovascular disease (CVD) [1]–[8]. Low-grade inflammation is accompanied by inflammatory cells that closely interact with the arterial wall, thereby driving the development of atherosclerosis and CVD [1], [2]. Because the role of low-grade inflammation in the pathophysiology of CVD is multi-faceted [1], [2], an extensive characterization by multiple biomarkers of low-grade inflammation is desirable. In this line, large cohort studies are increasingly adopting such a multiple biomarker approach [8]–[15].

Among a large variety of potential biomarkers [16], acute-phase reactants such as C-reactive protein (CRP) and serum amyloid A (SAA), and vascular adhesion molecules such as soluble intercellular adhesion molecule-1 (sICAM-1) and soluble vascular cell adhesion molecule-1 (sVCAM-1) reflect low-grade inflammation when present in low concentrations [1], [2], whereas high concentrations, such as CRP>10mg/l, more likely reflect acute inflammation or infection [9], [17], [18]. These four biomarkers of low-grade inflammation were studied because they play an important role in the pathophysiology of CVD [1], [2] and higher concentrations have been associated with (incident) CVD [3]–[8].

Traditionally, well-established analytical methods have enabled the analysis of single biomarkers of low-grade inflammation in one run. However, obtaining multiple biomarkers based on many single-biomarker measurements is very labor intensive, expensive and requires (relatively) large sample volumes. These limitations hamper an efficient multiple biomarker approach, particularly in large observational cohort or clinical trial studies. An attractive solution to these limitations is the simultaneous, and thus more efficient, measurement of a set of low-grade inflammatory biomarkers in one run. Such methods have recently become available with the use of multi-array platforms, such as the Luminex® and the MesoScaleDiscovery® (MSD) platforms and provide the tools necessary for efficient multiple biomarker detection. However, it remains to be established to what extent biomarker concentrations, as measured with these multi-array platforms, are comparable to well-established single-biomarker measurements. Although some cross-validation studies have been performed, most have not focused on biomarkers of low-grade inflammation [19]–[23] and the only study that did so pointed the problem of different measured concentrations, which may lead to bias in epidemiological associations [23].

Therefore, introducing a multi-array platform in the context of an ongoing longitudinal cohort study poses some challenges [24], [25] and cross-validation between methods within such a cohort is necessary before the ‘new’ method may replace the ‘old’ one. Specifically, one needs to determine whether substantial differences in biomarker concentrations are introduced by the new method, in which case realignment of the data by appropriate mathematical transformations may be required for the investigation of within-subjects changes in absolute concentrations of biomarkers over the course of time [26], [27]. In addition, the data obtained need also to be similarly associated with risk factors (RFs) known to be associated with low-grade inflammation to ensure that the multi-array platform measures what it intends to measure (i.e. face validity).

In view of these considerations, we compared the performance of a 4-plex multi-array electrochemiluminescense detection platform of low-grade inflammatory biomarkers (CRP, SAA, sICAM-1 and sVCAM-1) of MSD with that of well-established single-biomarker measurements, in a large ongoing cohort study of individuals with a wide spectrum of cardiovascular risk factors (RFs) known to be associated with low-grade inflammation.

## Materials and Methods

### Ethics Statement

The study was approved by the Medical Ethical Committee of the Maastricht University and all individuals gave written informed consent.

### Study Population and Design

The Cohort on Diabetes and Atherosclerosis Maastricht (CODAM) is a prospective cohort study that was originally designed to study the effects of obesity, glucose and lipid metabolism, lifestyle and genetics on cardiovascular complications, as described in detail elsewhere [28]–[33]. Briefly, individuals were selected from a large population-based cohort and included if they were of Caucasian ethnicity and older than 40 years, and met one or more of the following criteria: a body mass index (BMI) ≥25 kg/m^2^, a positive family history for type 2 diabetes mellitus, a history of gestational diabetes, use of anti-hypertensive medication, a postprandial glucose ≥6.0 mmol/l and/or glucosuria. In total, 574 individuals [mean age 59.6±7.0 years; 38.7% women] were included and extensively characterized with regard to their metabolic, cardiovascular and lifestyle risk profiles during 2 visits to the University research unit (CODAM-1, baseline examination: September 1999-July 2002). A first follow-up examination took place among 495 individuals (14% drop-out rate, mainly due to morbidity or mortality) approximately 7 years later (CODAM-2, July 2006-November 2009).

At baseline (i.e. CODAM-1), biomarkers of low-grade inflammation were assessed by single-biomarker techniques. At follow-up (i.e. CODAM-2), the single-biomarker techniques were replaced by the multi-array platform of MSD. To ensure comparability between methods, biomarkers of low-grade inflammation were also reassayed by the multi-array platform of MSD in all samples from the baseline examination (i.e. CODAM-1); at the time of these measurements, baseline samples had thus been stored for ∼7 years. The present cross-validation study reports on individuals’ paired data on biomarkers during the baseline examination (CODAM-1) and thus is a cross-sectional method comparison study. Method comparison for each biomarker was conducted on paired data, which were available for CPR in 566 individuals, for SAA in 563 individuals, for sICAM-1 in 566 individuals and for sVCAM-1 in 567 individuals and full paired data on all four inflammatory biomarkers were available in 550 individuals.

The CODAM study population is characterized by a wide spectrum of conditions known to be associated with low-grade inflammation [28]–[33]. Specifically, 52.4% of the individuals had normal glucose metabolism (NGM), 22.2% had impaired glucose metabolism (IGM) and 25.4% had type 2 diabetes mellitus (DM2) [28]–[33]. On the basis of measured BMI, the prevalence of normal weight, overweight and obesity was 18.2, 50.9 and 30.9%, respectively [32]. The metabolic syndrome, i.e. the clustering of ≥3 out of 5 cardiovascular RFs reflecting central obesity, dyslipidemia, elevated blood pressure and fasting plasma glucose, was prevalent in 54.7% of the study population [30], [31]. On the basis of self-reports, 29.3% of the individuals were never-smokers, 50.5% were ex-smokers and 20.2% were current smokers [33]. The prevalence of CVD was 27.6% and based on self-reports of myocardial infarction, coronary bypass surgery, stent placement or balloon dilatation, transient ischemic attack or stroke, abnormalities on a 12-lead electrocardiogram?(Minnesota codes 1.1 to 1.3, 4.1 to 4.3, 5.1 to 5.3 or 7.1) and(or) self-reported narrowing of peripheral arteries, non-traumatic limb amputation or a measured ankle-arm index <0.9 [30], [31]. Glycated hemoglobin [(mean ± SD) 6.0±0.82%] was measured as previously described [28]–[33]. Estimated glomerular filtration rate (eGFR) [(mean ± SD) 95.7±19.0 mL/min/1.73 m^2^] was calculated on the basis of individuals’ age, sex and serum creatinine levels according to the short Modification of Diet in Renal Disease (MDRD) equation [34].

### Biomarker Assessments

Individuals were asked to stop their lipid-lowering medication 14 days prior to the blood withdrawals and all other medication on the day before. After an overnight fast (duration of at least 10 hours) blood was drawn from the anticubital vein and collected in EDTA polypropylene tubes for plasma and in clot activator containing polypropylene tubes for serum. EDTA tubes were centrifuged at 3000 rpm for 15 min at 4°C, and plasma was immediately divided into 1 ml aliquots and stored in −80°C freezers until further analysis. Tubes with cloth activator were left 20 minutes before centrifugation at 3000 rpm for 15 min at 20°C, and serum was immediately divided into 1 ml aliquots and stored in −20°C freezers until analysis [28].

#### Biomarker detection by single-biomarker techniques

CRP was measured in a single measurement in serum with a high-sensitivity, immunoturbidimetric assay (detection range 100 ng/ml to 20000 ng/ml, i.e. factor 200) (Latex, Roche Diagnostics Netherlands BV, Almere, The Netherlands, www.roche.nl). This assay is based on the principle of particle-enhanced immunological agglutination. Briefly, anti-CRP antibodies coupled to latex micro-particles react with antigen present in the sample to form antigen-antibody complexes. Then, these micro-particles with antigen-antibody complexes agglutinate. This changes the fluid turbidity of the sample, which is detected by turbidimetry. sVCAM-1 was measured in EDTA plasma with a high-sensitivity human Quantikine ELISA kit (detection range 6.25 ng/ml to 200 ng/ml, i.e. factor 32) (R&D Systems, Minneapolis, MN, USA, www.rndsystems.com). sICAM-1 (detection range 0.625 ng/ml to 10 ng/ml, i.e. factor 16) and SAA (detection range 9.4 ng/ml to 600 ng/ml, i.e. factor 64) were measured in EDTA plasma by ELISA (Biosource, Invitrogen, Carlsbad, CA, USA, www.invitrogen.com). All low-grade inflammatory biomarkers were measured at the Laboratory of Toxicology, Genetics and Pathology of the National Institute for Public Health and the Environment, Bilthoven, The Netherlands [30]. The intra- and inter-assay coefficients of variation (CVs) for these assays were, for CRP, 0.6% and 1.9%; for SAA, 6.1% and 17.5%; for sICAM-1, 5.6% and 6.6%; and for sVCAM-1, 3.1% and 4.7%, respectively.

#### Biomarker detection by the 4-plex multi-array electrochemiluminescense detection platform of MesoScaleDiscovery

The 4-plex multi-array electrochemiluminescence platform of MesoScaleDiscovery (detection range 0.008 ng/ml to 1000 ng/ml, i.e. factor 125000) (MesoScaleDiscovery, Gaithersburg, MD, USA, www.mesoscale.com) was used to measure the four low-grade inflammatory biomarkers (CRP, SAA, sICAM-1 and sVCAM-1) simultaneously in EDTA plasma. This system uses multi-array plates fitted with multi-electrodes per well with each electrode being coated with a different capture antibody. For the present study the 4-plex assay (plates fitted with four electrodes per well, i.e. four separate well spots with a different capture antibody bound to each) was used. The assay procedure follows that of a classic sandwich ELISA with any of the analytes of interest captured on the relevant electrode. These captured analytes were, in turn, detected by a secondary, analyte-specific, ruthenium-conjugated antibody, which is capable of emitting light after electrochemical stimulation. This method minimizes nonspecific signals as the stimulation mechanism (electricity) is decoupled from the signal (light). According to the MSD protocol, each sample was analyzed in duplicate on the same array plate. All multi-array plates were analyzed within 16 days. The intra- and inter-assay CVs for the platform of MSD were, for CRP, 3.0% and 4.1%; for SAA, 2.5% and 11.8%; for sICAM-1, 2.5% and 4.7%; and, for sVCAM-1, 2.6% and 5.0%, respectively.

Variation between production lots of multi-array plates could influence biomarker measurements. We have evaluated the possible effect of lot-to-lot variation in the current 4-plex assay using additional data of previous studies [35], [36]. Based on biomarker data of 6 separate production lots (with an average of 30 plates per lot) the lot-to-lot CV for CRP was 9.8%, for SAA was 28.9%, for sICAM-1 was 3.4% and for sVCAM-1 was 4.9%. Thus, these variations were quite acceptable, except for SAA. Still, to avoid any noise due to lot-to-lot variation, all plasma samples of the CODAM study were measured within a single production lot of multi-array plates.

### Statistical Analyses

#### Method comparisons

Absolute concentrations of each biomarker as measured by the single-biomarker techniques and the multi-array platform were examined on all paired samples from the CODAM study baseline examination (n = 566 for CRP, n = 563 for SAA, n = 566 for sICAM-1 and n = 567 for sVCAM-1, after exclusion of erroneous outliers [37]). Pearson’s correlation coefficients were used to assess whether the ranking of each biomarker was similar between methods. Weighted Deming regression was used to assess the extent of constant and/or proportional bias between methods [26], [27]. This state-of-the-art statistical technique for method comparison is superior to simple linear regression by taking into account the error in both the dependent and independent variables [37], [38]. In addition, it allows random errors of each method to be proportional to the measured concentrations, such that the ratio of the CVs between methods remains constant over the concentration ranges (set at 1∶1 for regression calculations; e.g. 2% vs. 2% at low ranges, and 10% vs. 10% at high ranges) [37], [38].

#### Realignment and agreement

We anticipated that absolute biomarker concentrations, as obtained by either single- or multi-array methods, would differ due to a lack of standardization. Realignment of the data would, therefore, be necessary to enable direct comparison of absolute concentrations. For that purpose we used equations derived from Deming regression analyses to realign the data as obtained by one to the other method.

To examine the levels of agreement and verify the absence of systematic error *after* the re-alignment procedure, Bland-Altman plots of the differences between single-biomarker and multi-array data *vs.* their mean were obtained [39]. Bland-Altman plots were drawn on log*_e_* transformed data whenever the distribution of the differences was skewed [39], [40]. In addition, two-way mixed effects models (absolute agreement) were used to calculate intra-class correlation coefficients (ICC), which reflect similarity in individuals’ rank and similarity in absolute biomarker concentrations as obtained by single-biomarker techniques (*realigned)* and multi-array platform [41]. Note that the results of these analyses are shown in detail for single-biomarker data realigned to multi-array data for the following reason. The multi-array platform has recently been introduced in the CODAM study population and represents the methodology intended to carry on in follow-up assessments in this cohort.

#### Method performance across different cardiovascular risk groups

We used ANOVA to investigate the extent to which biomarker concentrations, either assessed by the single-biomarker techniques or the multi-array platform, increased across categories of glucose metabolism (i.e. NGM, IGM and DM2), weight (i.e. normal weight, overweight and obesity), number of traits of the metabolic syndrome (0–1 RFs, 2 RFs and ≥3 RFs) and smoking status (never, ex- and current-smoker), by appreciation of the group effects. ANOVA for repeated measures were subsequently used to ascertain whether such patterns of associations were similar between methods, by appreciation of group-by-method effects (the P-values of which should then be ≥0.05). In these analyses, (non-aligned) individual biomarker data, which are expressed in different scale units, were first standardized to comparable units by calculation of Z-scores as follows: (the individuals’ value – the population mean) \ the population SD. Per definition, each Z-score has a mean of 0, a SD of 1, and the same distribution as the absolute biomarker concentration (i.e. the ranking of individuals in the population remains the same). This thus enabled a direct comparison of the magnitude of relative differences in each biomarker by RF categories. All comparisons included adjustments for sex, age, eGFR and prior CVD and were conducted among individuals with complete paired data on all four biomarkers (n = 550).

All analyses were performed with the use of the Statistical Package for Social Sciences (SPSS Inc, version 15.0, Chicago, Illinois, USA, www.spss.com), except weighted Deming regression, which was analyzed using the Analyse-It software (Analyse-it Software Ltd, Leeds, UK, www.analyse-it.com) for Microsoft Excel (Microsoft Corporation, Washington, USA, www.microsoft.com). Statistical significance was set at a P-value <0.05.

## Results

### Biomarker Concentrations

Table 1 shows the absolute concentrations of CRP, SAA, sICAM-1, and sVCAM-1, as measured with the single-biomarker techniques or the multi-array platform, in the whole study population and across RFs categories.

**Table 1:** Absolute biomarker concentrations in the total population and according to glucose metabolism, weight, metabolic syndrome and smoking status as determined by the single-biomarker techniques or by the multi-array platform of MesoScaleDiscovery.

	CRP (mg/l)		SAA (mg/l)		sICAM-1 (µg/l)		sVCAM-1 (µg/l)	
	Immunoturbidimetry	Multi-array	ELISA	Multi-array	ELISA	Multi-array	ELISA	Multi-array
Total population (n = 550)	2.6 [1.4–4.5]	1.9 [0.9–3.8]	7.0 [4.0–13.8]	1.3 [0.8–2.2]	350±91	219±54	476±121	339±75
***Glucose metabolism status*** a								
NGM (n = 291)	2.2 [1.3–3.7]	1.6 [0.9–3.1]	6.2 [3.7–11.8]	1.2 [0.7–2.0]	338±85	210±50	463±121	331±73
IGM (n = 122)	2.8 [1.5–4.8]	2.1 [1.0–3.9]	8.0 [4.5–14.9]	1.3 [0.9–2.6]	354±90	220±48	467±105	338±68
DM2 (n = 137)	3.2 [1.9–5.7]	2.4 [1.3–5.3]	8.1 [4.7–15.5]	1.5 [1.0–2.6]	373±100	237±63	510±127	356±82
***Weight status*** b								
Normal weight (n = 100)	1.4 [0.9–3.0]	1.0 [0.5–2.4]	5.3 [3.0–12.0]	1.0 [0.6–1.9]	326±99	205±50	454±130	323±70
Overweight (n = 283)	2.2 [1.4–3.8]	1.6 [0.9–3.1]	6.4 [4.2–12.6]	1.2 [0.8–2.0]	341±79	214±47	467±114	334±73
Obese (n = 167)	3.6 [2.3–6.0]	3.0 [1.6–5.4]	8.6 [5.2–15.8]	1.6 [1.0–2.7]	380±98	238±62	505±121	357±76
***Metabolic syndrome status*** c								
0–1 risk factor (n = 134)	1.5 [0.9–3.1]	1.0 [0.5–2.4]	5.6 [3.4–11.6]	1.1 [0.7–1.8]	317±75	198±42	447±106	322±65
2 risk factors (n = 119)	2.4 [1.3–3.8]	1.8 [0.9–3.2]	6.5 [4.0–14.7]	1.3 [0.8–2.5]	334±80	205±41	464±117	324±67
≥3 risk factors d (n = 297)	3.0 [1.8–5.2]	2.4 [1.3–4.7]	7.7 [4.5–14.2]	1.4 [0.9–2.4]	372±96	235±58	494±126	352±79
***Smoking status***								
Never (n = 161)	2.1 [1.3–3.4]	1.5 [0.9–2.7]	6.1 [3.8–13.6]	1.3 [0.8–2.1]	331±82	211±49	485±125	337±75
Ex-smoker (n = 278)	2.6 [1.4–4.6]	2.0 [0.9–3.9]	6.8 [4.2–12.6]	1.3 [0.8–2.0]	347±88	217±57	481±117	344±73
Current (n = 111)	3.2 [1.5–5.5]	2.4 [1.0–5.3]	9.4 [4.5–15.6]	1.5 [0.9–2.7]	385±101	237±50	450±122	327±78

Data are means ± SD or medians [interquartile range].

a NGM, normal glucose metabolism: defined as fasting plasma glucose <6.1 mmol/l and 2-hour post-load plasma glucose <7.8 mmol/l; IGM, impaired glucose metabolism: includes impaired fasting plasma glucose (between 6.1 mmol/l and 7.0 mmol/l) and/or impaired glucose tolerance (2-hour post-load plasma glucose between 7.8 and 11.1 mmol/l); DM2, diabetes mellitus type 2 (fasting plasma glucose ≥7.0 mmol/l and/or 2-hour post-load plasma glucose ≥11.1 mmol/l);

b Categorized on the basis of individuals’ body mass index (BMI) as: normal (if BMI 18.5–24.9 kg/m^2^); overweight (if BMI ≥25.0 and <29.9 kg/m^2^), and obese (if BMI ≥30 kg/m^2^);

c Metabolic syndrome status was defined according to the revised NCEP-ATPIII definition (American Heart Association/National Heart, Lung and Blood Institute);

d any 3 out of the following traits/risk factors reflect the presence of the syndrome: elevated waist circumference (≥102 cm in men, ≥88 cm in women); reduced HDL-cholesterol (<1.03 mmol/l in men, <1.29 mmol/l in women, and/or specific drug treatment); elevated triglycerides (≥1.7 mmol/l and/or specific drug treatment); elevated blood pressure (systolic/diastolic ≥130/85 mm Hg and/or anti-hypertensive treatment); and elevated fasting plasma glucose (≥5.6 mmol/l and/or glucose-lowering treatment); CRP, C-reactive protein; SAA, serum amyloid A; sICAM-1, soluble intercellular adhesion molecule 1; sVCAM-1, soluble vascular cell adhesion molecule 1.

#### Method comparison

**Figure 1:**
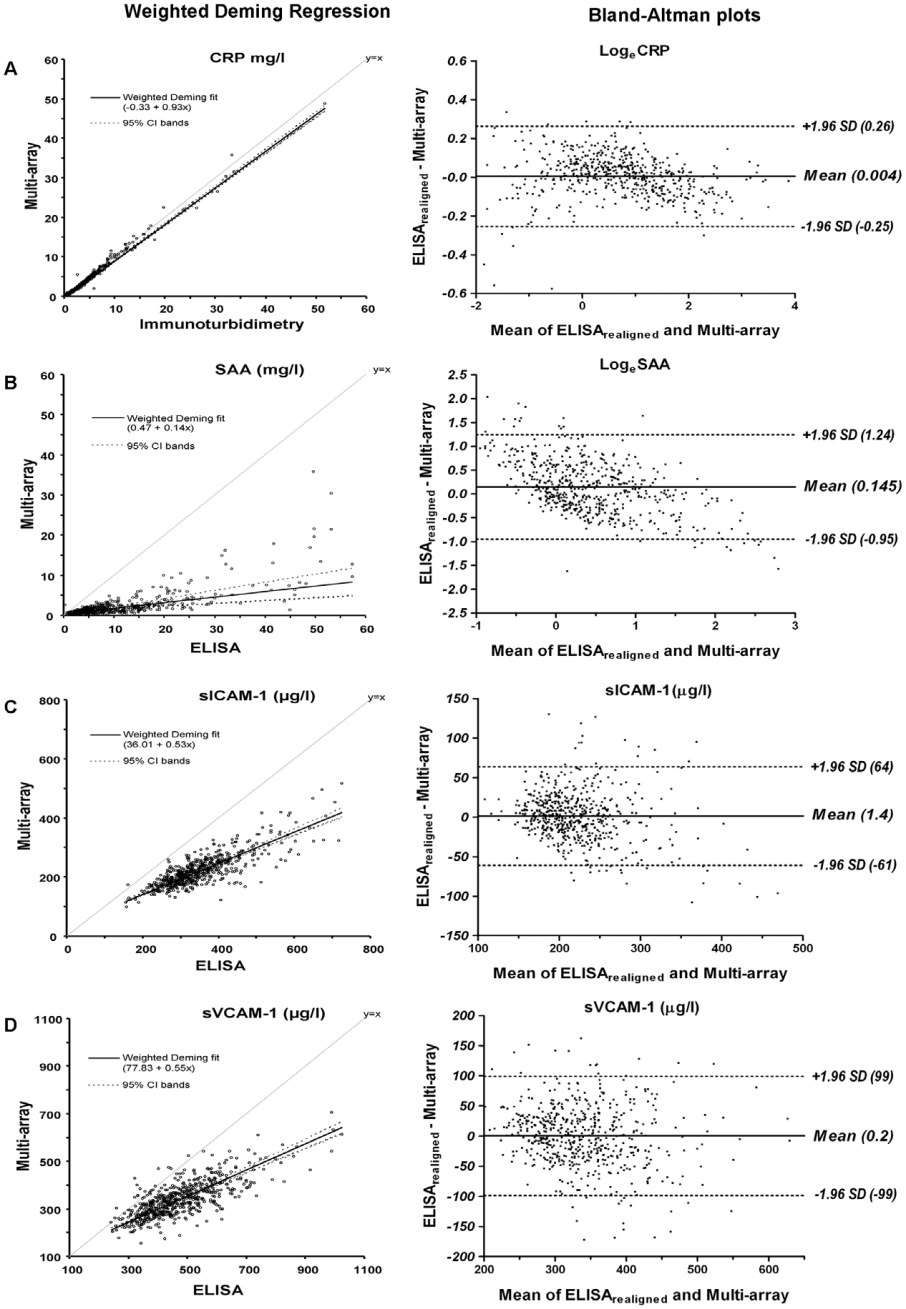
Left panel: comparison of biomarker concentrations between the single-biomarker techniques and the multi-array platform by weighted Deming regression; Right panel: Bland-Altman plots of the differences between the two methods. (**A**) CRP, C-reactive protein (n = 566); (**B**) SAA, serum amyloid A (n = 563); (**C**) sICAM-1, soluble intercellular adhesion molecule 1 (n = 566); and (**D**) sVCAM-1, soluble vascular cell adhesion molecule 1 (n = 567). For log*_e_* transformed data limits of agreement can be converted to original units by anti-log calculation (e.g., for CRP, e^−0.25^ and e^0.26^ which are equivalent to 0.78 and 1.30 times or 22% below or 30% above the long axis of CRP, respectively).

Despite the very high Pearson’s correlation coefficients (i.e. 0.994 for CRP, 0.758 for SAA, 0.816 for sICAM-1 and 0.755 for sVCAM-1) absolute concentrations of biomarkers as obtained by single-biomarker vs. multi-array techniques differed considerably. Indeed, weighted Deming regression analyses for all biomarkers showed significant constant (intercepts) and proportional (slopes) bias between methods such that the absolute mean concentrations of all four biomarkers were lower when measured with the multi-array platform than with the single-biomarker techniques (Fig. 1A–D, left panels). The above indicates that, when comparing *absolute* values, realignment of the single-biomarker data to the multi-array data (or vice-versa) is thus warranted.

#### Realignment and agreement

**Figure 2:**
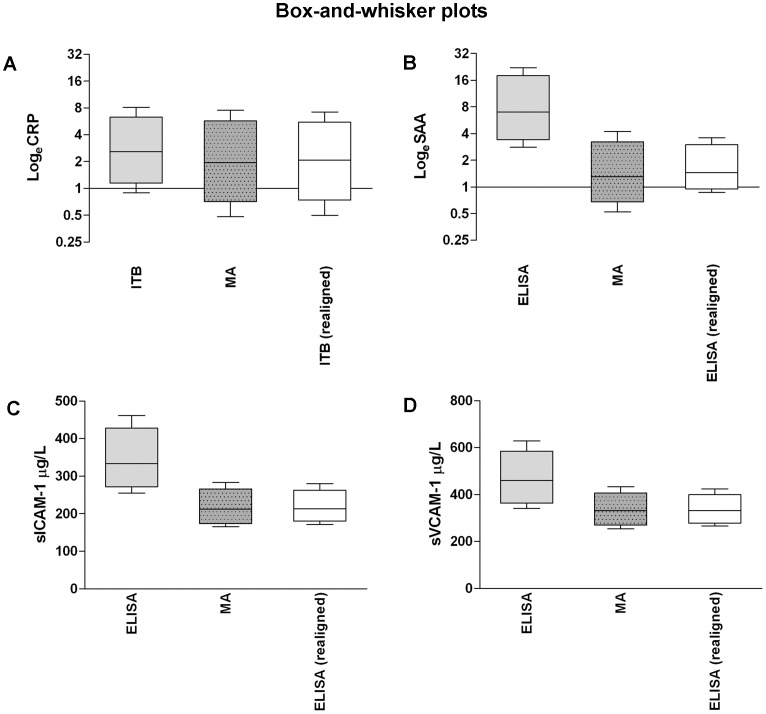
Box-and-whisker plots showing median and interquartile (box) and 10^th^ and 90^th^ centiles (whiskers) for each biomarker, before and after realignment to current (multi-array) concentrations. (**A**) CRP, C-reactive protein (n = 566); (**B**) SAA, serum amyloid A (n = 563); (**C**) sICAM-1, soluble intercellular adhesion molecule 1 (n = 566); and (**D**) sVCAM-1, soluble vascular cell adhesion molecule 1 (n = 567); ITB, immunoturbidimetry; MA, multi-array.

Realignment of the data as obtained by different methods was therefore conducted with the use of the coefficients retrieved from the Deming regression models (Table 2). Bland-Altman plots of the single-biomarker data *realigned* to the multi-array data (Fig. 1A–D, right panels) showed that no obvious relation of differences between methods with their mean was present. For all biomarkers, except SAA, Bland-Altman plots confirmed the removal of systematic bias after the realignment (all mean values for differences between methods around 0 (Fig. 1A, 1C and 1D, right panels)). For SAA, a systematic difference between ELISA and multi-array data still persisted after the realignment (about 15%, i.e. e^0.145^ as compared to their mean (Fig. 1B, right panel)). In addition, the equations applied for the *realignment* (Table 2) resulted in similar distributions of single-biomarker and multi-array data (Fig. 2). The resulting ICCs between single-biomarker (*realigned*) and multi-array data were 0.996 for CRP, 0.711 for SAA, 0.895 for sICAM-1 and 0.858 for sVCAM-1.

**Table 2:** Weighted Deming regression.

		Regression equations (weighted Deming)^a^						
Variable	N pairs	Y	X	intercept		slope		Sylx
				α	95% CI	β	95% CI	
CRP (mg/l)	566	CRP_MA_	CRP_ITB_	−0.33	−0.35; −0.31	0.93	0.91; 0.94	0.102
SAA (mg/l)	563	SAA_MA_	SAA_ELISA_	0.47	0.05; 0.90	0.14	0.07; 0.20	0.239
sICAM-1 (µg/l)	566	sICAM-1_MA_	sICAM-1_ELISA_	36.01	24.63; 47.39	0.53	0.49; 0.56	0.104
sVCAM-1 (µg/l)	567	sVCAM-1_MA_	sVCAM-1_ELISA_	77.83	58.90; 96.75	0.55	0.51; 0.59	0.124
CRP (mg/l)	566	CRP_ITB_	CRP_MA_	0.35	0.34; 0.37	1.08	1.06; 1.10	0.110
SAA (mg/l)	563	SAA_ELISA_	SAA_MA_	−3.46	−7.37; 0.45	7.33	4.40; 10.25	1.751
sICAM-1 (µg/l)	566	sICAM-1_ELISA_	sICAM-1_MA_	−68.49	−94.71; −42.27	1.90	1.77; 2.03	0.197
sVCAM-1 (µg/l)	567	sVCAM-1_ELISA_	sVCAM-1_MA_	−141.52	−186.54; −96.49	1.82	1.68; 1.96	0.225

a Data are intercepts (α) and (slopes) β of the Deming regression equation, which all differed significantly from 0 and 1, respectively, as indicated by their 95% CI; rejection of the hypothesis that α = 0 means that the two methods differ at least by a constant amount; rejection of the hypothesis that β = 1 implies that there is a proportional difference between methods; Sylx are standard deviations of the residuals;

Upper panel: these equations are used as cross-validation equations to realign single-biomarker data (ITB, immunoturbidimetry; or ELISA) to multi-array (MA) data.

Lower panel: these equations are used as cross-validation equations to realign multi-array (MA) data to single-biomarker data (ITB, immunoturbidimetry; or ELISA).

CRP, C-reactive protein; SAA, serum amyloid A; sICAM-1, soluble intercellular adhesion molecule 1; sVCAM-1, soluble vascular cell adhesion molecule 1.

#### Method performance across different cardiovascular risk groups

Concentrations of all biomarkers, as measured by single-biomarker or multi-array methods (expressed as Z-scores), increased significantly across categories of glucose metabolism, weight, metabolic syndrome and smoking status (all P-trends ≤0.028, except for sVCAM-1 and smoking status), independently of sex, age, eGFR and prior CVD (Table 3). Importantly, the patterns of associations between RFs level and individual biomarker concentrations did not differ by method of detection [all P-values for group*method interaction were >0.05, except for metabolic syndrome status and Log_e_CRP (P-value  = 0.002)] (Table 3).

**Table 3:** Comparison of individual biomarker Z-scores according to glucose metabolism, weight, metabolic syndrome and smoking status as determined by the single-biomarker techniques or by the multi-array platform of MesoScaleDiscovery *^a.^*

	Log*_e_*CRP					Log*_e_*SAA				sICAM-1				sVCAM-1			
	ITB		Multi-array			ELISA		Multi-array		ELISA		Multi-array		ELISA		Multi-array	
	mean	95% CI	mean		95% CI	mean	95% CI	mean	95% CI	mean	95% CI	mean	95% CI	mean	95% CI	mean	95% CI
***Glucose metabolism status***																	
NGM (n = 291)	−0.13	−0.24; −0.01		−0.13	−0.24; −0.02	−0.11	−0.23; 0.00	−0.09	−0.20; 0.02	−0.11	−0.23; 0.00	−0.14	−0.26; −0.03	−0.09	−0.20; 0.02	−0.08	−0.20; 0.03
IGM (n = 122)	0.06	−0.12; 0.23		0.06	−0.11; 0.24	0.11	−0.06; 0.28	0.001	−0.17; 0.17	0.05	−0.13; 0.22	0.01	−0.16; 0.19	−0.09	−0.26; 0.09	−0.02	−0.19; 0.15
DM2 (n = 137)	0.21	0.05; 0.38		0.22	0.05; 0.39	0.14	−0.02; 0.31	0.20	0.03; 0.36	0.20	0.03; 0.37	0.29	0.13; 0.46	0.26	0.10; 0.42	0.20	0.03; 0.36
P-value for linear trend	0.001			0.001		0.014		0.005		0.003		*<0.001*		0.001		0.007	
P-value interaction group*method	*0.655*					*0.146*				*0.152*				*0.357*			
***Weight status***																	
Normal weight (n = 100)	−0.53	−0.71; −0.34		−0.55	−0.74; −0.37	−0.28	−0.47; −0.09	−0.28	−0.47; −0.09	−0.24	−0.44; −0.05	−0.25	−0.44; −0.06	−0.17	−0.36; 0.02	−0.20	−0.39; −0.006
Overweight (n = 283)	−0.06	−0.17; 0.05		−0.05	−0.16; 0.06	−0.04	−0.15; 0.07	−0.07	−0.18; 0.04	−0.09	−0.20; 0.03	−0.09	−0.20; 0.02	−0.09	−0.20; 0.03	−0.07	−0.19; 0.04
Obese (n = 167)	0.41	0.27; 0.56		0.42	0.28; 0.56	0.23	0.09; 0.38	0.28	0.14; 0.43	0.29	0.14; 0.44	0.30	0.15; 0.45	0.24	0.10; 0.39	0.24	0.10; 0.39
P-value for linear trend	*<0.001*			*<0.001*		*<0.001*		*<0.001*		*<0.001*		*<0.001*		*0.001*		*<0.001*	
P-value interaction group*method	*0.185*					*0.567*				*0.952*				*0.881*			
***Metabolic syndrome status***																	
0–1 risk factors (n = 134)	−0.39	−0.56; −0.23		−0.43	−0.59; −0.26	−0.17	−0.34; 0.00	−0.16	−0.32; 0.01	−0.33	−0.49; −0.16	−0.36	−0.52; −0.20	−0.19	−0.36; −0.03	−0.18	−0.34; −0.01
2 risk factors (n = 119)	−0.09	−0.26; 0.08		−0.09	−0.26; 0.08	−0.02	−0.19; 0.16	−0.002	−0.18; 0.17	−0.18	−0.35; −0.001	−0.26	−0.43; −0.09	−0.12	−0.29; 0.05	−0.21	−0.38; −0.03
≥3 risk factors (n = 297)	0.21	0.10; 0.32		0.23	0.12; 0.34	0.08	−0.03; 0.20	0.07	−0.04; 0.18	0.22	0.11; 0.33	0.27	0.16; 0.38	0.14	0.02; 0.25	0.16	0.05; 0.27
P-value for linear trend	*<0.001*			*<0.001*		*0.016*		*0.028*		*<0.001*		*<0.001*		*0.001*		*0.001*	
P-value interaction group*method	*0.002*					*0.922*				*0.103*				*0.322*			
***Smoking status***																	
Never (n = 161)	−0.23	−0.39; −0.08		−0.24	−0.40; −0.09	−0.17	−0.33; −0.02	−0.14	−0.29; 0.02	−0.22	−0.38; −0.07	−0.18	−0.34; −0.03	0.07	−0.08; 0.22	−0.03	−0.18; 0.13
Ex-smoker (n = 278)	0.07	−0.05; 0.18		0.06	−0.06; 0.18	0.03	−0.08; 0.15	−0.001	−0.12; 0.11	−0.02	−0.14; 0.10	−0.03	−0.14; 0.09	0.01	−0.10; 0.13	0.05	−0.07; 0.16
Current (n = 111)	0.18	−0.01; 0.36		0.20	0.01; 0.38	0.17	−0.02; 0.35	0.20	0.02; 0.38	0.38	0.19; 0.56	0.33	0.14; 0.51	−0.13	−0.31; 0.05	−0.08	−0.26; 0.11
P-value for linear trend	*0.001*			*<0.001*		*0.006*		*0.007*		*<0.001*		*<0.001*		*0.101*		*0.687*	
P-value interaction group*method	*0.205*					*0.543*				*0.489*				*0.144*			

Data are means of standardized biomarker concentrations (Z-scores); all data are adjusted for sex, age, estimated glomerular filtration rate and prior CVD;

NGM, normal glucose metabolism: IGM, impaired glucose metabolism; DM2, diabetes mellitus type 2; CRP, C-reactive protein; SAA, serum amyloid A; sICAM-1, soluble intercellular adhesion molecule 1; sVCAM-1, soluble vascular cell adhesion molecule 1; ITB, immunoturbidimetry.

These results did not materially change, when the analyses were repeated excluding individuals with CRP values >10 mg/l, likely to indicate an acute inflammatory response [9], [17], [18] (data not shown).

### Additional Analyses

A key step in biochemical tests comparison is to ascertain whether the level of agreement between methods is acceptable from a clinical standpoint [40]. For CRP, values <1, 1–3, and >3 mg/l have been proposed to identify individuals at low, intermediate and high-risk for incident CVD, respectively, whereas such values are lacking for the other biomarkers examined herein. This impairs the appreciation of the clinical relevance of the limits of agreement between methods obtained for these biomarkers (Fig. 1B–D, right panels) [8], [9]. Still, for CRP we could ascertain that, on the basis of immunoturbidimetry, 12.9% of the CODAM Study population would be classified at ‘low risk’, 46.4% at ‘intermediate risk’ and 40.7% at ‘high-risk’; on the basis of the multi-array platform these numbers would be 28.0%, 39.5% and 32.5%, respectively (Cohen’s κ = 0.641, which is a measure of agreement for categorical data; overall concordance rate of 76.7%). After realignment of the immunoturbidimetry to the multi-array data and vice versa, the agreement between methods increased considerably (Cohen’s κ of 0.931 and 0.946 and concordance rates of 95.4 and 96.7%, respectively - Table 4).

**Table 4:** Agreement in risk level assignment on the basis of CRP obtained by immunoturbidimetry and the multi-array platform.

CRP		Multi-array platform						Multi-array platformrealigned to Immunoturbidimetry					
	Level	<1 mg/l(%)	1–3 mg/l (%)	>3 mg/l(%)	Total(%)	Concordance(%)	κ	<1 mg/l(%)	1–3 mg/l (%)	>3 mg/l(%)	Total(%)	Concordance(%)	κ
Immunoturbidimetry	<1 mg/l	**12.9**	0.0	0.0	12.9		0.641	**12.5**	0.4	0.0	12.9		0.946
	1–3 mg/l	15.1	**31.3**	0.0	46.4			0.5	**45.5**	0.4	46.4		
	>3 mg/l	0.0	8.2	**32.5**	40.7			0.0	2.0	**38.7**	40.7		
	Total	28.0	39.5	32.5	100.0			13.1	47.8	39.1	100.0		
						**76.7**						**96.7**	
Immunoturbidimetry realigned to multi-array	<1 mg/l	**26.0**	0.4	0.0	26.4		0.931	–	–	–	–		–
	1–3 mg/l	2.0	**37.6**	0.7	40.4			–	–	–	–		
	>3 mg/l	0.0	1.5	**31.8**	33.3			–	–	–	–		
	Total	28.0	39.5	32.5	100.0			–	–	–	–		
						**95.4**						–	

κ, Cohen’s kappa (measure of agreement for categorical data); –, not applicable.

## Discussion

The present study has three main findings. First, the absolute concentrations of CRP, SAA, sICAM-1 and sVCAM-1 differed significantly between the single-biomarker techniques and the multi-array platform of MSD. Second, equations retrieved by weighted Deming regression enabled proper realignment of the data to overcome these absolute differences. Finally, the overall pattern of associations between levels of the individual biomarkers with glucose metabolism, weight, metabolic syndrome and smoking status did not differ by method of detection. This is the first study that has examined and cross-validated, in a large ongoing cohort study, measurements of biomarkers of low-grade inflammation by means of single-biomarker techniques and the multi-array platform of MSD.

Our results are in line with a previous study, which suggested that data measured with single-biomarker techniques and data measured with the multi-array platform cannot be combined without appropriate realignment of the data as this would distort epidemiological associations [23]. In our study, the absolute concentrations of all four biomarkers were lower when measured with the multi-array platform than with the single-biomarker techniques. It should be emphasized, however, that the absolute concentration of each biomarker is based on the standards provided by the commercial kits and the lack of international standardization among these may therefore explain the differences between methods [9]. Although CRP reference materials exist, bias attributed to standardization remains due to the fact that reference materials were developed to distinguish between CRP values below 10 mg/l, from 10 to 40 mg/l and above 40 mg/l, whereas current assays aim for accurate and reproducible detection down to 0.3 mg/l [18]. Also according to the Centers for Disease Control and Prevention and the American Heart Association laboratory science discussion group, further standardization efforts are therefore required as measurements of absolute biomarker concentrations are of paramount importance for direct comparison between studies using different methods and for definition of clinical cutoff values [18]. Nevertheless, in the present study we were able to appropriately realign the data to overcome the absolute differences between both methods. Thus, the introduction of a multi-array platform in an ongoing cohort study may be implemented without impairing the investigation of within-subject changes in biomarker concentrations over the course of time. This was enabled by re-assaying all the baseline samples with the new multi-array method. In addition, we show that the agreement in risk level assignment on the basis of CRP levels (<1, 1–3, and >3 mg/l [8], [9]) is very high after realignment. It remains, however, that subjects’ risk-level assignment depends on the method used for CRP assessment, and that if this were done on the basis of MSD readings, less individuals from the CODAM Study would be considered to be at high-risk than if this were done on the basis of immunoturbidimetry readings. However, to establish which method is superior in risk prediction further studies are warranted.

Another option to directly compare individual biomarker levels between methods (but also between clinical studies) is by transformation of data to Z-scores, especially if realignment equations are lacking. By Z-score transformation, between-subjects ranking in terms of their biomarkers levels are preserved within the population. The present study shows that Z-scores of CRP, SAA, sICAM-1, sVCAM-1 differed across categories of glucose metabolism, weight, metabolic syndrome and smoking status in a similar fashion irrespective of the method of detection. Although it is evident that a high correlation between assays will result in identical associations, these results, illustrate and emphasize that, despite absolute differences, the relative differences are comparable between the single-biomarker techniques and the multi-array platform.

Taken together, our findings suggest that the multi-array platform of MSD could potentially replace the single-biomarker techniques for the detection of multiple biomarkers in large ongoing and future clinical studies aiming at the investigation of the role of low-grade inflammation in the etiology of CVD, though careful validation would be required.

Furthermore, the multi-array platform of MSD has several practical advantages over the well-established single-biomarker techniques for biomarker detection, although CRP assays are generally automated [18]: 1) it has simple operating procedures; 2) it has a higher sensitivity and greater detection range, which eliminates multiple dilutions and freeze and thaw cycles per sample; 3) it allows determination of four (or more) biomarkers simultaneously, improving the labor-efficiency, and due costs; and 4) it uses a small sample volume (5 µL instead of 50 µL for the detection of these four markers), which is useful in clinical and epidemiological studies.

The present study has some limitations. First, with the single-biomarker techniques, CRP was measured in serum and SAA, sVCAM-1 and sICAM-1 were measured in plasma, whereas with the multi-array platform all biomarkers were measured in plasma. This may, in part, explain the differences between methods in absolute concentrations of CRP, since a different matrix might effect detection. Furthermore, the measurement of biomarkers by the single-biomarker techniques and the multi-array platform were performed ∼7 years apart, which could also have contributed to an underestimation of absolute biomarker concentrations by the multi-array platform. However, because storage time of samples was the same for all study individuals, if anything: 1) this underestimation was likely systematic and properly incorporated in the realignment equations; and 2) could not have affected the relative differences in biomarkers across different levels of subjects’ cardiovascular RFs. Second, we showed realignment equations to enable transition of ‘old’ to ‘new’ methods within our ongoing cohort study (and vice versa). However, the results were shown in detail for single-biomarker data realigned to multi-array data. This way of presentation facilitates future comparisons of those biomarkers measured with the multi-array platform at follow-up examinations within this ongoing cohort study. However, any other cohort study should calculate realignment equations within their own data. These may be susceptible to lot-to-lot variation, although in our laboratory the lot-to-lot variation between multi-array assays was low for most of the biomarkers. Nevertheless, the measured concentrations will always depend on the standards provided by the commercial kits (for both the single biomarker and multi-array techniques), which have not been satisfactorily standardized internationally [9], [18].

In conclusion, multiple biomarker detection by the 4-plex multi-array platform of MSD including CRP, SAA, sICAM-1 and sVCAM-1 shows comparable results with well-established single-biomarker techniques, despite differences in absolute concentrations. Subjects’ risk-level assignment therefore depends on the method used. It is, however, uncertain which method is superior in risk prediction. Nevertheless, these biomarkers of low-grade inflammation are associated with glucose metabolism, weight, metabolic syndrome and smoking status, irrespective of the method of detection. In terms of time, effort and quality, this multi-array platform of MSD is an attractive alternative for single-biomarker measurements. Therefore, this platform is a potential tool for the quantification of multiple biomarkers of low-grade inflammation using small sample volume in one single run in large ongoing and future clinical studies.

## References

[1] Borissoff JI, Spronk HM, ten Cate H (2011) The hemostatic system as a modulator of atherosclerosis. N Engl J Med 364: 1746–1760. 21542745 10.1056/NEJMra1011670

[2] Ross R (1999) Atherosclerosis–an inflammatory disease. N Engl J Med 340: 115–126. 9887164 10.1056/NEJM199901143400207

[3] Becker A, van Hinsbergh VW, Jager A, Kostense PJ, Dekker JM, et al (2002) Why is soluble intercellular adhesion molecule-1 related to cardiovascular mortality? Eur J Clin Invest 32: 1–8. 10.1046/j.1365-2362.2002.00919.x 11851720

[4] Blankenberg S, Rupprecht HJ, Bickel C, Peetz D, Hafner G, et al (2001) Circulating cell adhesion molecules and death in patients with coronary artery disease. Circulation 104: 1336–1342. 11560847 10.1161/hc3701.095949

[5] Kaplan RC, McGinn AP, Baird AE, Hendrix SL, Kooperberg C, et al (2008) Inflammation and hemostasis biomarkers for predicting stroke in postmenopausal women: the Women's Health Initiative Observational Study. J Stroke Cerebrovasc Dis 17: 344–355. 18984425 10.1016/j.jstrokecerebrovasdis.2008.04.006 PMC3077422

[6] Ridker PM, Hennekens CH, Buring JE, Rifai N (2000) C-reactive protein and other markers of inflammation in the prediction of cardiovascular disease in women. N Engl J Med 342: 836–843. 10733371 10.1056/NEJM200003233421202

[7] Tzoulaki I, Murray GD, Lee AJ, Rumley A, Lowe GD, et al (2007) Relative value of inflammatory, hemostatic, and rheological factors for incident myocardial infarction and stroke: the Edinburgh Artery Study. Circulation 115: 2119–2127. 17404162 10.1161/CIRCULATIONAHA.106.635029

[8] Vasan RS (2006) Biomarkers of cardiovascular disease: molecular basis and practical considerations. Circulation 113: 2335–2362. 16702488 10.1161/CIRCULATIONAHA.104.482570

[9] Pearson TA, Mensah GA, Alexander RW, Anderson JL, Cannon RO, 3rd, et al (2003) Markers of inflammation and cardiovascular disease: application to clinical and public health practice: A statement for healthcare professionals from the Centers for Disease Control and Prevention and the American Heart Association. Circulation 107: 499–511. 12551878 10.1161/01.cir.0000052939.59093.45

[10] Schnabel RB, Larson MG, Yamamoto JF, Kathiresan S, Rong J, et al (2009) Relation of multiple inflammatory biomarkers to incident atrial fibrillation. Am J Cardiol 104: 92–96. 19576326 10.1016/j.amjcard.2009.02.053 PMC2802058

[11] Conen D, Ridker PM, Everett BM, Tedrow UB, Rose L, et al (2010) A multimarker approach to assess the influence of inflammation on the incidence of atrial fibrillation in women. Eur Heart J 31: 1730–1736. 20501475 10.1093/eurheartj/ehq146 PMC2903714

[12] Kim HC, Greenland P, Rossouw JE, Manson JE, Cochrane BB, et al (2010) Multimarker prediction of coronary heart disease risk: the Women's Health Initiative. J Am Coll Cardiol 55: 2080–2091. 20447530 10.1016/j.jacc.2009.12.047

[13] Daniels LB, Maisel AS (2010) Multiple marker approach to risk stratification in patients with stable coronary artery disease: to have or have not. Eur Heart J 31: 2980–2983. 20864490 10.1093/eurheartj/ehq336

[14] Wang TJ (2010) Multiple biomarkers for predicting cardiovascular events: lessons learned. J Am Coll Cardiol 55: 2092–2095. 20447531 10.1016/j.jacc.2010.02.019

[15] Gruson D, Bodovitz S (2010) Rapid emergence of multimarker strategies in laboratory medicine. Biomarkers 15: 289–296. 20100037 10.3109/13547500903560065

[16] Tedgui A, Mallat Z (2006) Cytokines in atherosclerosis: pathogenic and regulatory pathways. Physiol Rev 86: 515–581. 16601268 10.1152/physrev.00024.2005

[17] Thanabalasingham G, Shah N, Vaxillaire M, Hansen T, Tuomi T, et al (2010) A large multi-centre European study validates high-sensitivity C-reactive protein (hsCRP) as a clinical biomarker for the diagnosis of diabetes subtypes. Diabetologia 54: 2801–2810. 10.1007/s00125-011-2261-y 21814873

[18] Myers GL, Rifai N, Tracy RP, Roberts WL, Alexander RW, et al (2004) CDC/AHA Workshop on Markers of Inflammation and Cardiovascular Disease: Application to Clinical and Public Health Practice: report from the laboratory science discussion group. Circulation 110: e545–549. 15611379 10.1161/01.CIR.0000148980.87579.5E

[19] Marchese RD, Puchalski D, Miller P, Antonello J, Hammond O, et al (2009) Optimization and validation of a multiplex, electrochemiluminescence-based detection assay for the quantitation of immunoglobulin G serotype-specific antipneumococcal antibodies in human serum. Clin Vaccine Immunol 16: 387–396. 19158284 10.1128/CVI.00415-08 PMC2650878

[20] Oh ES, Mielke MM, Rosenberg PB, Jain A, Fedarko NS, et al (2010) Comparison of conventional ELISA with electrochemiluminescence technology for detection of amyloid-beta in plasma. J Alzheimers Dis 21: 769–773. 20634583 10.3233/JAD-2010-100456 PMC3075593

[21] Prabhakar U, Eirikis E, Davis HM (2002) Simultaneous quantification of proinflammatory cytokines in human plasma using the LabMAP assay. J Immunol Methods 260: 207–218. 11792390 10.1016/s0022-1759(01)00543-9

[22] Dupont NC, Wang K, Wadhwa PD, Culhane JF, Nelson EL (2005) Validation and comparison of luminex multiplex cytokine analysis kits with ELISA: determinations of a panel of nine cytokines in clinical sample culture supernatants. J Reprod Immunol 66: 175–191. 16029895 10.1016/j.jri.2005.03.005 PMC5738327

[23] de Koning L, Liptak C, Shkreta A, Bradwin G, Hu FB, et al.. (2012) A multiplex immunoassay gives different results than singleplex immunoassays which may bias epidemiologic associations. Clin Biochem doi:10.1016/j.clinbiochem.2012.1004.1006. 10.1016/j.clinbiochem.2012.04.006 22537455

[24] Steffes M, Cleary P, Goldstein D, Little R, Wiedmeyer HM, et al (2005) Hemoglobin A1c measurements over nearly two decades: sustaining comparable values throughout the Diabetes Control and Complications Trial and the Epidemiology of Diabetes Interventions and Complications study. Clin Chem 51: 753–758. 15684277 10.1373/clinchem.2004.042143 PMC2635091

[25] Cull CA, Manley SE, Stratton IM, Neil HA, Ross IS, et al (1997) Approach to maintaining comparability of biochemical data during long-term clinical trials. Clin Chem 43: 1913–1918. 9342012

[26] Maahs DM, Jalal D, McFann K, Rewers M, Snell-Bergeon JK (2011) Systematic shifts in cystatin C between 2006 and 2010. Clin J Am Soc Nephrol 6: 1952–1955. 21784814 10.2215/CJN.11271210 PMC3156426

[27] Selvin E, Coresh J, Zhu H, Folsom A, Steffes MW (2010) Measurement of HbA1c from stored whole blood samples in the Atherosclerosis Risk in Communities study. J Diabetes 2: 118–124. 20923494 10.1111/j.1753-0407.2010.00070.x PMC2991637

[28] Kruijshoop M, Feskens EJ, Blaak EE, de Bruin TW (2004) Validation of capillary glucose measurements to detect glucose intolerance or type 2 diabetes mellitus in the general population. Clin Chim Acta 341: 33–40. 14967156 10.1016/j.cccn.2003.10.033

[29] Du H, van der AD, van Bakel MM, van der Kallen CJ, Blaak EE, et al (2008) Glycemic index and glycemic load in relation to food and nutrient intake and metabolic risk factors in a Dutch population. Am J Clin Nutr 87: 655–661. 18326604 10.1093/ajcn/87.3.655

[30] Jacobs M, van Greevenbroek MM, van der Kallen CJ, Ferreira I, Blaak EE, et al (2009) Low-grade inflammation can partly explain the association between the metabolic syndrome and either coronary artery disease or severity of peripheral arterial disease: the CODAM study. Eur J Clin Invest 39: 437–444. 19397692 10.1111/j.1365-2362.2009.02129.x

[31] Jacobs M, van Greevenbroek MM, van der Kallen CJ, Ferreira I, Blaak EE, et al (2010) The association between the metabolic syndrome and peripheral, but not coronary, artery disease is partly mediated by endothelial dysfunction: the CODAM study. Eur J Clin Invest 41: 167–175. 21039444 10.1111/j.1365-2362.2010.02392.x

[32] Thewissen MM, Damoiseaux JG, Duijvestijn AM, van Greevenbroek MM, van der Kallen CJ, et al (2010) Abdominal Fat Mass Is Associated With Adaptive Immune Activation: The CODAM Study. Obesity (Silver Spring) 19: 1690–1698. 10.1038/oby.2010.337 21253003

[33] van Greevenbroek MM, Jacobs M, van der Kallen CJ, Blaak EE, Jansen EH, et al (2012) Human plasma complement C3 is independently associated with coronary heart disease, but only in heavy smokers (the CODAM study). Int J Cardiol 154: 158–162. 20926148 10.1016/j.ijcard.2010.09.017

[34] Levey AS, Coresh J, Greene T, Marsh J, Stevens LA, et al (2007) Expressing the Modification of Diet in Renal Disease Study equation for estimating glomerular filtration rate with standardized serum creatinine values. Clin Chem 53: 766–772. 17332152 10.1373/clinchem.2006.077180

[35] van Bussel BC, Schouten F, Henry RM, Schalkwijk CG, de Boer MR, et al (2011) Endothelial dysfunction and low-grade inflammation are associated with greater arterial stiffness over a 6-year period. Hypertension 58: 588–595. 21859964 10.1161/HYPERTENSIONAHA.111.174557

[36] van Bussel BC, Henry RM, Schalkwijk CG, Dekker JM, Nijpels G, et al (2012) Low-grade inflammation, but not endothelial dysfunction, is associated with greater carotid stiffness in the elderly: the Hoorn Study. J Hypertens 30: 744–752. 22343535 10.1097/HJH.0b013e328350a487

[37] Linnet K (1998) Performance of Deming regression analysis in case of misspecified analytical error ratio in method comparison studies. Clin Chem 44: 1024–1031. 9590376

[38] Martin RF (2000) General deming regression for estimating systematic bias and its confidence interval in method-comparison studies. Clin Chem 46: 100–104. 10620577

[39] Bland JM, Altman DG (1986) Statistical methods for assessing agreement between two methods of clinical measurement. Lancet 1: 307–310. 2868172

[40] Twomey PJ (2006) How to use difference plots in quantitative method comparison studies. Ann Clin Biochem 43: 124–129. 16536914 10.1258/000456306776021616

[41] de Vet HC, Terwee CB, Knol DL, Bouter LM (2006) When to use agreement versus reliability measures. J Clin Epidemiol 59: 1033–1039. 16980142 10.1016/j.jclinepi.2005.10.015

